# Gene expression atlas of the Colorado potato beetle (*Leptinotarsa decemlineata*)

**DOI:** 10.1038/s41597-025-04607-7

**Published:** 2025-02-19

**Authors:** Léonore Wilhelm, Yangzi Wang, Shuqing Xu

**Affiliations:** 1 Institute of Organismic and Molecular Evolution (iomE), Johannes Gutenberg University, 55128 Mainz, Germany; 2 Institute for Evolution and Biodiversity, University of Münster, 48161 Münster, Germany

**Keywords:** Genetic databases, Evolutionary genetics, Invasive species, Entomology, Gene expression

## Abstract

The Colorado potato beetle (CPB) is a major pest of potato crops, known for its remarkable ability to develop resistance to more than 50 pesticides. For decades, CPB has served as a model species for studying insecticide resistance, insect physiology, diapause, reproduction, and evolution. However, research progress on CPB has been hindered by the lack of comprehensive genomic and transcriptomic resources. Here, leveraging a recently established chromosome-level genome assembly, we constructed a gene expression atlas of CPB using transcriptomic data from 61 samples representing major organs and developmental stages. By integrating short- and long-read sequencing technologies, we enhanced the genome annotation and identified 6,623 additional genes that were previously undetected. Furthermore, we developed a web portal to facilitate the search and visualization of the gene expression atlas, providing an accessible resource for the research community. The CPB gene expression atlas offers valuable tools and comprehensive data that will accelerate future research in pest control and insect biology.

## Background & Summary

The Colorado potato beetle (CPB) is a devastating pest originating from North America that has spread in Europe and Asia in the 20^th^ century. It causes substantial damage to solanaceous plants, in particular potatoes, as one CPB can consume around 40 cm^2^ of leaf tissue during the larval stages and 100 cm^2^ leaf area every 10 days during its adult life^1^. The CPB exhibits remarkable resistance to pesticides. More than 50 active compounds proved to be ineffective against various tested populations (Mota-Sanchez and Wise 2017 Arthropod Pesticide Resistance Database). Studies have shown that resistance to many insecticides evolved rapidly in response to the applications of insecticides and showed geographical variation^2–6^. This is largely due to high genetic variation in the population^7^, likely as the result of CPB’s high fecundity. A mated female CPB can produce 25 eggs per day on average^8^ and up to 724 eggs in 30 days^9^, generating a large pool of individuals upon which selection can act. Adaptation to pesticides appears to be polygenic, involving genes related to detoxification, cuticle composition, and neuronal receptors^7,10^.

The CPB has been the object of extended studies, most of which are related to understanding the mechanisms of pesticide resistance. Agronomic studies on the CPB include bioassays with potential pesticides^11,12^, use of biocontrol agents^13^, and RNA interference^14–17^. Recent studies have focused on finding alleles linked with resistance^18,19^ and gene expression linked with resistance^20^. The CPB has also been used as a model to understand insect development and physiology^21–23^, insect diapause^24–27^, and more recently insect lipid metabolism and calcium signalling^28,29^ as well as behaviour^30^.

Despite the abundance of research topics addressed using the CPB, the research in CPB is currently constrained by the lack of accessible genomic and transcriptomic data. The chromosome-level reference genome has been released recently and several studies on population genomics of CPB have been carried out^31,32^. Yet, we currently have no publicly available gene expression data among tissues and developmental stages, which is vital for understanding the genetic mechanisms of most phenotypic traits in CPB. Here, we sequenced the transcriptomes of 12 tissues in adults (five from female, seven from male), five tissues in the last instar larvae, as well as the eggs and entire body of each larval stage. Using these data, we further improved the genome annotation and established a gene expression atlas of CPB (Table 1). The updated genome annotation contains 34,350 genes with a BUSCO score of 96.2%, which is 3% higher than the previous annotation. To provide easy access to the expression data, we established a web-based portal allowing users to search, download and visualize the expression of genes among organs or developmental stages. The CPB atlas can be accessed via https://cpb-atlas.uni-mainz.de/.

**Table 1:** Comparison of the functional annotation from Yan *et al*. with the annotation from this paper.

Annotation version	Data used	Gene number	Busco score
The i5k Initiative	• 13 RNA-seq samples of whole-body of adults, larvae and tissues. • Protein homologs from the Insecta GenBank database and 7 RefSeq proteomes	16533	C:93.9%[S:92.2%,D:1.7%],F:3.3%,M:2.7%,n:2124
Yan *et al*.^31^	• 13 RNA-seq samples of whole-body of adults and 1^st^ instar larvae • Protein homologs of all insects downloaded from OrthoDB	27692	C:93.5%[S:86.0%,D:7.5%],F:1.0%,M:5.5%,n:1367
This paper	• 17 RNA-seq samples from different tissues and developmental stages (Supplementary Table 2) • Protein homologs from *D. melanogaster* and *T. castaneum* downloaded from Uniprot • Long reads from 21 sample types • Genes annotated in Yan *et al*. but absent in our current annotation were added to the annotation.	34315	C:96.2%[S:88.1%,D:8.1%],F:0.9%,M:2.9%,n:1367

## Methods

**Fig. 1:**
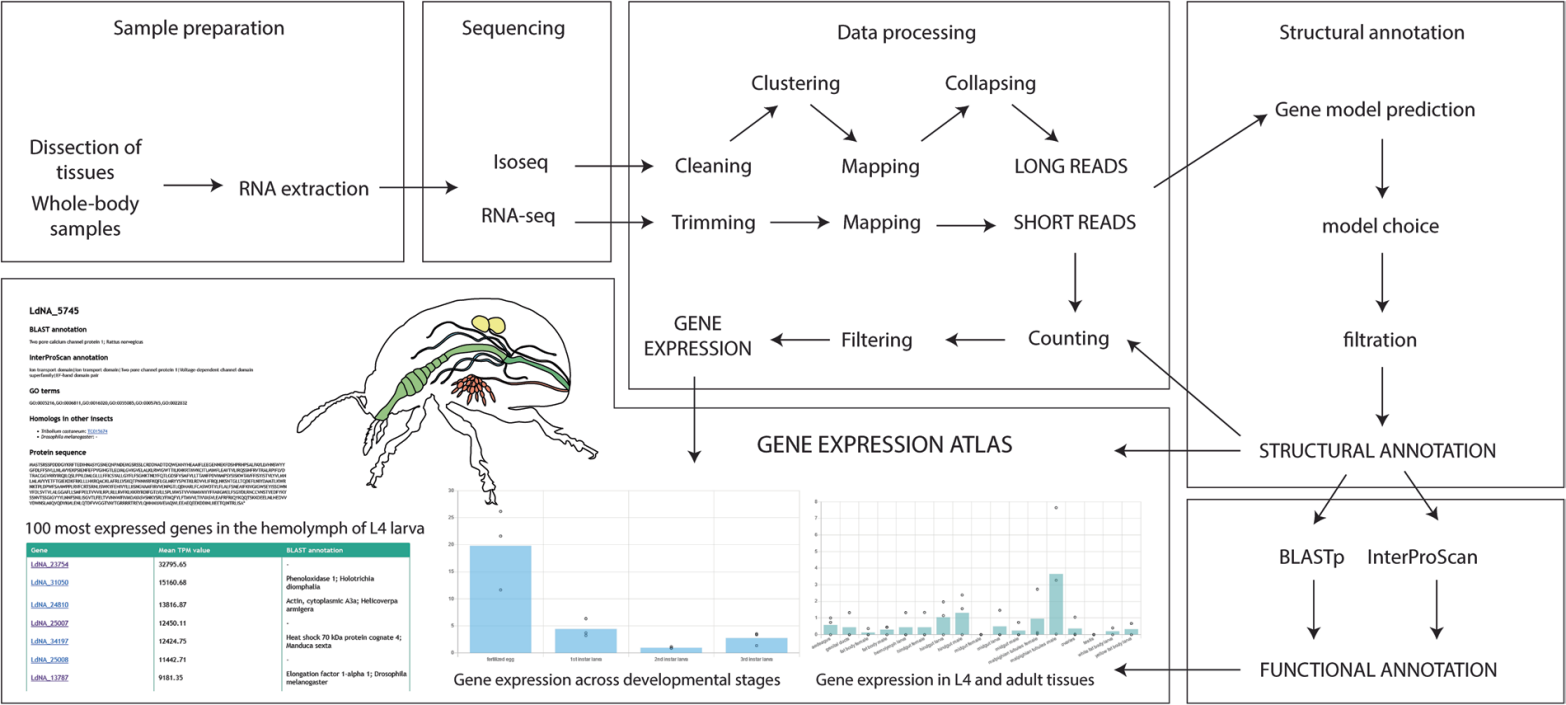
Graphical representation of the CPB atlas workflow. The samples were first prepared (either dissected or directly frozen), RNA was extracted, then sequenced. The data from RNA-seq and Isoseq were processed separately. Both were used to construct the structural annotation i.e. position of the genes and their exons and CDS on the genome assembly. The structural annotation was employed later to build the functional annotation i.e. function of each gene, by a search with BLASTp and InterProScan separately. The short reads were mapped to the structural annotation to obtain an expression count for the genes. The resulting gene expression were displayed on a web portal in the form of bar plots.

The insects were maintained in a climate chamber with 24 °C, 70% humidity and a photoperiod of 16 h light and 8 h dark per day. Larvae, as well as adults, were fed with leaves from organically grown potato plants (Annabelle variety, purchased from Ellenberg’s Kartoffelvielfalt GmbH & Co. KG, Barum, Germany), similar to our previous work^30^. The E06 strain, which was originally collected from Spain in 2012, was used for all samples^33^. The experimental design comprises the following steps (Fig. 1): dissection of the specimens, RNA extraction, RNA sequencing, data processing, and generation of the functional annotations.

### Dissection and RNA extraction

**Fig. 2:**
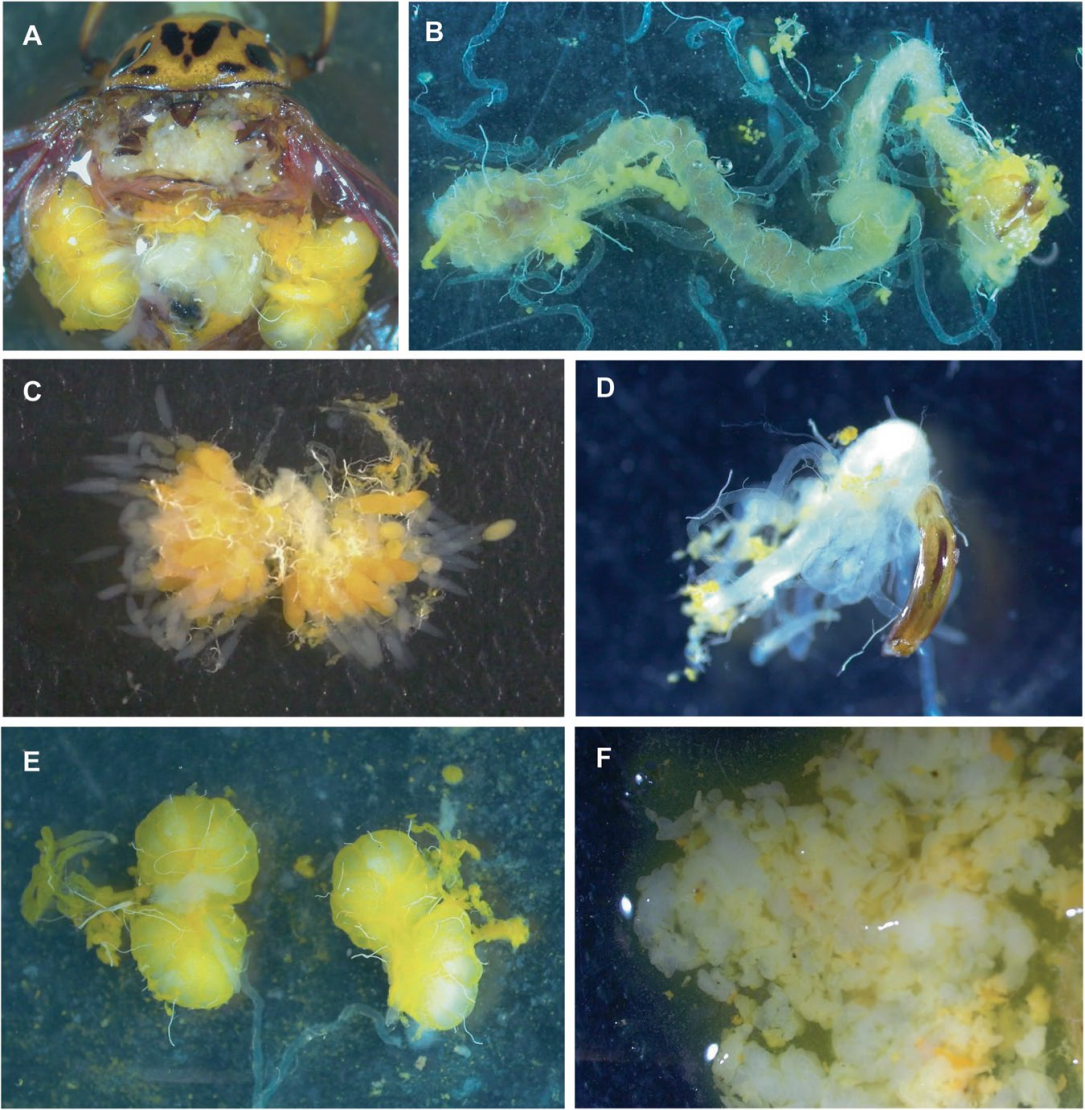
Dissection of tissues: (**A**) Male incised dorsally. (**B**) Midgut, hindgut and malpighian tubules; (**C**) Ovaries; (**D**) Aedeagus and genital ducts; (**E**) Testes; (**F**) Fat body (white and yellow) of a fourth instar.

The CPB atlas includes transcriptomes of tissues from fourth instar larvae (L4) and adults, as well as the whole body from different stages, from egg to third instar larva (Fig. 2). The tissues were dissected in PBS under a stereoscopic microscope. Dissection time was limited to 10 min to avoid RNA degradation, and the tissues were subsequently snap-frozen in liquid nitrogen and stored in a −80 °C freezer. The whole-body samples were similarly snap-frozen before being stored to ensure identical processing of the entire dataset. The eggs were pooled into groups of five. Three L1, L2, and L3 larvae were pooled to create each sample, respectively. We pooled individuals because the extracted RNA from each individual was not sufficient for sequencing. The L4 larvae and adult tissues are from single individuals. The L4 larvae were dissected on the 19^th^ day of development from egg laying. Adults were kept in individual petri dishes from emergence and dissected seven days later. RNA was extracted using the RNeasy Mini kit from Qiagen (Venlo, The Netherlands), following the manufacturer’s protocol. Lysis was performed with micro pestles. To obtain the full-length transcript of all the genes using an iso-seq approach, we pooled 200 ng RNA from one replicate of each sample type (except two due to their low RNA abundance; Supplementary table 1). The 61 RNA-seq samples and one iso-seq sample were sequenced using Illumina NovaSeq and PacBio Sequel sequencers, respectively, at Novogene (Cambridge, UK).

For RNA-seq library preparation, the Novogene NGS RNA Library Prep Set (PT042) was used. The magnetic beads of oligos d(T)25 were used to enrich the mRNA. Subsequently, mRNA was randomly fragmented, and cDNA synthesis was carried out using random hexamers and the reverse transcriptase enzyme. The second chain was synthesized with the addition of an Illumina buffer (non-directional library preparation). The resulting products were purified, end-repaired, and ligated with adapters. Fragments of the appropriate size are enriched by PCR, where indexed P5 and P7 primers are introduced, and final products are purified. The libraries were checked with Qubit 2.0 and real-time PCR for quantification and bioanalyzer Agilent 2100 for size distribution detection. Quantified libraries will be pooled and sequenced on the Illumina Novaseq. 6000 platform, according to effective library concentration and data amount, using the paired-end 150 strategy (PE150).

For Iso-seq, the SMRTbell library was prepared. The first strand cDNA was synthesized from the polyA tail by NEBNext’s reverse transcriptase. Then the enzyme’s terminal transferase activity added a few extra nucleotides to the 3′ end of the cDNA. Subsequently, template switching, extension and amplification were performed to generate double-stranded cDNA. The cDNA was ligated with universal hairpin adapters. After purification with magnetic beads, the sequencing primer was annealed to the SMRTbell templates, followed by binding of the sequencing polymerase to the annealed templates. The SMRTbell library was sequenced in the PacBio Sequel IIe platform, in CCS mode. We used three replicates for each sample except the white fat body and the yellow fat body of the L4 larva, for which two samples were used.

### Iso-seq data processing

Iso-seq data were processed using the command-line tools from PacBio’s SMRT Link software (available at https://www.pacb.com/). First, consensus reads were generated with the function *css*. We demultiplexed reads with *lima* and then removed polyA tails with *isoseq3 refine* using default parameters. The reads were clustered with *isoseq3 cluster*. The Full-Length Non-Concatemer (FLNC) reads obtained were mapped to the genome^31^ using pbmm2, the pacbio wrapper for minimap2^34^. Finally, they were collapsed using Cupcake (v0.1.4, https://github.com/Magdoll/cDNA_Cupcake) to produce non-redundant full-length transcripts.

### RNA-seq data processing

Trimmomatic was used to remove the Illumina adapters, drop the reads with a low quality or a short length (SLIDINGWINDOW:4:15 MINLEN:36) and remove the first ten bases of the reads (HEADCROP:10). To assess the quality both before and after this filtering process, we utilized FastQC (v0.11.9, https://www.bioinformatics.babraham.ac.uk/projects/fastqc/). The reads were mapped to a chromosome-level genome assembly^31^ using STAR^35^ (version 2.7.8a). FeatureCounts (Subread 2.0.5) was used to count the reads mapped to the annotated genes, with the parameters “-p -countReadPairs” which indicates that the reads are paired. The genes that were not expressed were filtered out based on their transcript per million (TPM) values. We kept only the transcript having a TPM value above one in at least two samples (https://github.com/Xu-lab-Evolution/CPB_gene_expression_atlas/blob/main/TPMfiltration.R). After filtration, 15,578 genes out of 34,350 were kept in the gene expression atlas.

### Protein-coding gene annotation

Prior to genome annotation, we removed the contaminants using FCS GX^36^ (v 0.5.0, https://github.com/ncbi/fcs-gx). For the prediction of protein-coding genes, we employed a modified BRAKER^37–41^ pipeline (https://github.com/Gaius-Augustus/BRAKER/tree/master). In brief, this approach integrates gene models predicted based on both short-read RNA-seq transcriptome (BRAKER1 method^42–47^) and protein homologs from *Drosophila melanogaster* and *Tribolium castaneum* (BRAKER2 method^40,43,44,48–52^). Subsequently, TSEBRA^41^ was used to compare these predictions against full-length transcripts obtained from Iso-seq data, thereby determining the most accurate gene models.

 When running BRAKER1, we combined paired-end RNA-seq reads from 17 different tissue types (Supplementary table 2) and used the repeat soft-masked CPB reference genome^31^. The short reads facilitated the automatic training of GeneMark-ET by using the spliced alignment data to assist gene model predictions by AUGUSTUS^53^. For BRAKER2, a similar process was conducted for the automatic training of GeneMark-EP+^52^. However, in this step, BRAKER2 employed information on protein-coding exon boundaries derived from the alignment of homologous protein sequences from *Drosophila melanogaster* and *Tribolium castaneum* (downloaded from UniProt^54^) to assist AUGUSTUS in gene prediction. We used GeneMarkS-T^55^ to identify the protein-coding regions within each full-length transcript. We then subsequently merged gene models from BRAKER1 and BRAKER2 and compared them against the Iso-seq full-length transcripts using TSEBRA. We retained only the longest isoform for each gene model. Using AGAT (v0.9.0, https://github.com/NBISweden/AGAT) we removed genes that are shorter than 100 bp and single-exon genes that lacked complete start or stop codons. Unique gene models that were present in the previous annotation version^31^ and had no overlap with any models predicted in the current version were retained in the new annotation. For the functional annotations, protein sequences were aligned to the UniProtKB^20^ database using *blastp* (BLAST + v2.12.0)^44^ with “-evalue 1e-6 -max_hsps 1 -max_target_seqs 1 -outfmt 6”. The results were further processed using InterProScan (5.71–102.0)^56^ with the options “-goterms -iprlookup”. The KEGG pathways were added using BlastKOALA^57^.

### Weighted gene co-expression network analysis

We performed a weighted gene co-expression network analysis using the WGCNA package in R^58^. The data were normalized using counts adjusted by TMM factors (CTF), we then applied a hyperbolic arcsine (asinh) transformation following the procedure described by Johnson and Krishnan^59^. We used tissue samples from adults and L4 larvae and kept genes with TPM values greater than one in at least one sample. The network was constructed with the blockwiseModules function and the parameters TOMType = “signed” and power = 9. The genes were divided into 52 modules; the smaller one (Salmon4) contained 20 genes and the largest (Turquoise) contained 3,002 genes. We performed correlation analysis to link modules eigengenes to tissues using the Pearson coefficient (Supplementary figure 3).

### Tissue-specificity of the genes

Tissue-specificity of the genes has been tested with the extended tau score^60,61^. This score attributes a value from 0 (ubiquitous) to 1 (specific) to each gene of the dataset. We first calculated the median TPM value for each gene in every tissue. Those values were log_2_ transformed, and the tau score was calculated using the formula described in Yanai *et al*.^60^. The genes with a tau score greater than 0.85 and with a median TPM value above 10 in all tissues were considered specific. These genes were then assigned tissues using the extended tau score method, which determines a threshold based on the maximum expression value, the standard deviation and a Z-value derived from a Fuzzy c-means clustering of lower and upper bounds expression.

A total of 2487 genes (16% of the expressed genes) were tissue-specific, amongst which 2,246 were specific to one tissue, 227 to two tissues, and 14 to three tissues. The testes were by far the tissue presenting the more tissue-specific genes, with a number of 1,102 genes. Oppositely, the fat body displayed only 61 specific genes.

### Gene Ontology enrichment analysis

To check whether the RNA-seq data accurately reflects the transcriptome of each tissue, a gene ontology enrichment analysis was performed on the tissue-specific genes identified previously. The Enricher function of the ClusterProfiler package on R was used separately on the GO terms belonging to the “molecular function” and the “biological process” ontologies^62^. The results suggest that the GO terms enriched among tissue-specific genes are related to the function of each tissue (Supplementary table 3). For example, testes-specific genes were, amongst others, enriched in the biological processes of cilium assembly (GO:0060271) and cilium movement (GO:0003341). The fat body specific genes were enriched in fatty acid biosynthetic process (GO:0006633) and long-chain fatty-acyl-CoA metabolic process (GO:0035336).

## Data Records

The raw data and the analysis results have been deposited in Figshare^63^. The raw sequencing data are also available in the NCBI Sequence Read Archive (SRA) under accession numbers SAMN39526582 to SAMN39526643, associated with BioProject PRJNA1067435. The expression data can be accessed under GEO accession number GSE285883^64^. The genome annotation, the transcriptome and the various analysis results are available on the web application: https://cpb-atlas.uni-mainz.de/resources.

## Technical Validation

**Fig. 3:**
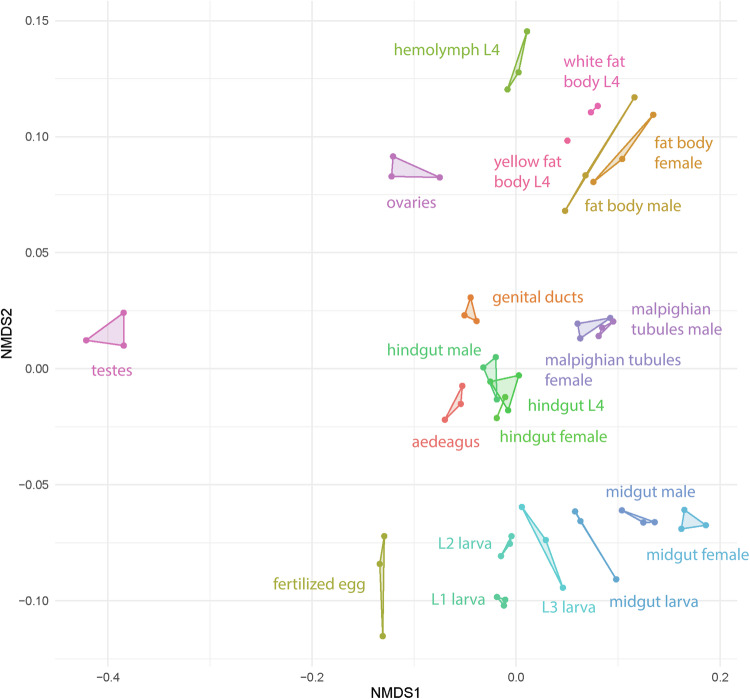
Non-metric multidimensional scaling (NMDS) of the samples based on log_2_(TPM + 1) values. Biological replicates are connected.

**Fig. 4:**
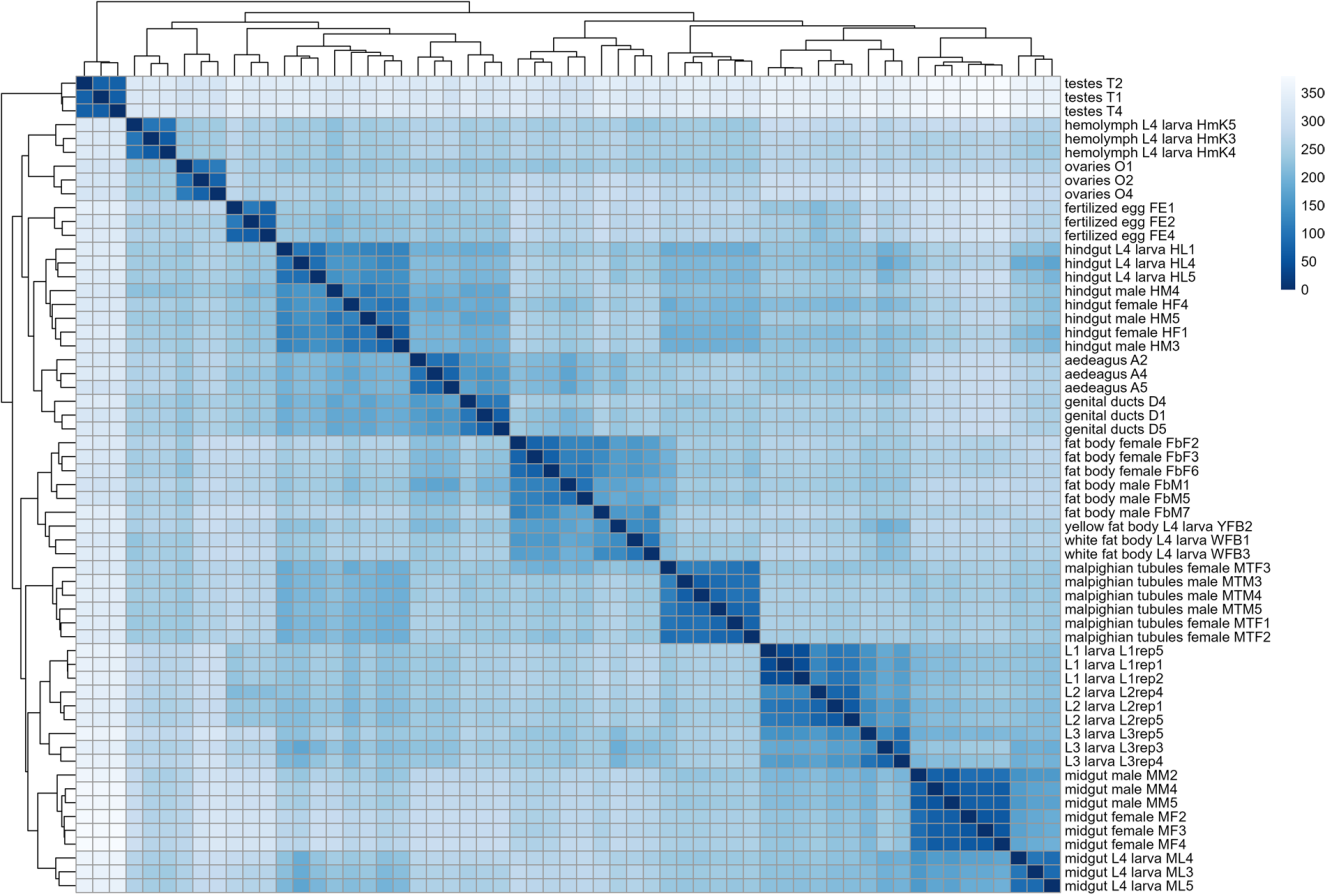
Heatmap showing the pairwise comparisons of log_2_(TPM) values across the 59 samples of the gene expression atlas. All the tissues are correctly clustering together showing an absence of contaminations from other tissues. Some tissues also segregate by developmental stage (e.g. midgut of L4 larvae vs adults) showing a modification of the transcriptome through age. On the contrary there are no clear separation of the tissues between the sexes in adult, showing the absence of sex-specific transcriptome for tissues common to both sexes.

The sample integrity, purity, and quantification were verified using an Agilent 5400 device. Samples were visualized on an NMDS and a pairwise heatmap to detect potential outliers using the log_2_(TPM + 1) values (Supplementary figures 1, 2). Two outliers were detected: a yellow fat body and a female hindgut. As this is likely due to contamination during dissection and sample collection steps, we removed these two samples from the CPB atlas. After the removal of outliers, all the visualization methods were applied again and showed that replicates cluster closely to each other (Figs. 3, 4).

## Supplementary information

Supplementary files

## Data Availability

The scripts used to process short reads are available at the address: https://github.com/Xu-lab-Evolution/CPB_gene_expression_atlas/releases/tag/release_v1.0. The genome annotation has been generated using the same method described at https://github.com/Xu-lab-Evolution/Waterlily_aphid_genome_project^65^.
