# Supplementary material for: Gene expression atlas of the Colorado potato beetle (Leptinotarsa decemlineata)
Source: Sci Data. 2025 Feb 19;12:299. doi: 10.1038/s41597-025-04607-7 (PMC11840028; doi:10.1038/s41597-025-04607-7)
Supplement: Supplementary file 1 — Supplementary files [file 41597_2025_4607_MOESM1_ESM.pdf]

Supplementary table 1. Samples for which an extract have been collected and pooled for long read sequencing.

RNA-seq ID	Tissue types
A1	aedeagus
D2	genital ducts
FbF6	fat body female
FBM7	fat body male
FE2	fertilized egg
HF5	hindgut female
HL1	hindgut larva
HM5	hindgut male
HmK2	hemolymph
ML4	midgut larva
MM5	midgut male
MTF1	malpighian tubule female
MTM3	malpighian tubule male
O4	ovaries
T2	testis
L1_1	whole-body first instar larva
L2_1	whole-body second instar larva
L3_2	whole-body third instar larva

Supplementary table 2. Samples provided to BRAKER to build the structural annotation.

RNA-seq ID	Tissue types
A2	aedeagus
D1	genital ducts
FbF2	fat body female
FBM1	fat body male
FE1	fertilized egg
HF1	hindgut female
HL1	hindgut larva
HM3	hindgut male
HmK3	hemolymph
ML3	midgut larva
MM2	midgut male
MTF1	malpighian tubule female
MTM3	malpighian tubule male
O1	ovaries
T1	testis
WFB1	white fat body larva
YFB2	yellow fat body larva

Supplementary table 3. Enrichment of the GO terms belonging to the "Biological Process" ontology in the tissues of the atlas. Each row corresponds to one GO term (second column) significantly enriched in one tissue (first column). The third column corresponds to the ratio of genes with this GO term specific to that tissue, to the total number of genes specific to that tissue. The fourth column corresponds to the number of genes containing this GO term to the total number of genes containing a "Biological Process" GO term. The fifth column is the adjusted p-value and the sixth, the name of the GO term.

tissue	go_id	gene_ratio	bg_ratio	p_adj	name
aedeagus	GO:0006629	8/53	78/9251	3.12124943710647e-07	lipid metabolic process
aedeagus	GO:0019752	3/53	17/9251	0.00154025375201827	carboxylic acid metabolic process
aedeagus	GO:0006979	3/53	37/9251	0.0108212661129219	response to oxidative stress
aedeagus	GO:0021556	2/53	13/9251	0.0162849707112457	central nervous system formation
aedeagus	GO:0006032	2/53	20/9251	0.0309314390522668	chitin catabolic process
aedeagus	GO:0006520	2/53	29/9251	0.0399742163441064	amino acid metabolic process
aedeagus	GO:0016042	2/53	29/9251	0.0399742163441064	lipid catabolic process
aedeagus	GO:0045087	2/53	29/9251	0.0399742163441064	innate immune response
fat body	GO:0006633	6/23	24/9251	2.27920925807676e-10	fatty acid biosynthetic process
fat body	GO:0035336	5/23	30/9251	6.12259787169019e-08	long-chain fatty-acyl-CoA metabolic process
fat body	GO:0009058	4/23	37/9251	9.0813677086112e-06	biosynthetic process
fat body	GO:0019367	3/23	18/9251	2.67301652539312e-05	fatty acid elongation, saturated fatty acid
fat body	GO:0034625	3/23	18/9251	2.67301652539312e-05	fatty acid elongation, monounsaturated fatty acid
fat body	GO:0034626	3/23	18/9251	2.67301652539312e-05	fatty acid elongation, polyunsaturated fatty acid
fat body	GO:0042761	3/23	22/9251	4.29602219748673e-05	very long-chain fatty acid biosynthetic process

fat body	GO:0030148	3/23	23/9251	4.3158637511002e-05	sphingolipid biosynthetic process
genital ducts	GO:0071805	6/47	57/9251	1.21816630208891e-05	potassium ion transmembrane transport
genital ducts	GO:0030182	4/47	40/9251	0.000771309023561292	neuron differentiation
genital ducts	GO:0042391	3/47	17/9251	0.000800418185438304	regulation of membrane potential
genital ducts	GO:0006355	9/47	377/9251	0.000800418185438304	regulation of DNA-templated transcription
genital ducts	GO:0006813	3/47	22/9251	0.00116748537512978	potassium ion transport
genital ducts	GO:0030322	2/47	17/9251	0.0180015961797593	stabilization of membrane potential
genital ducts	GO:0006032	2/47	20/9251	0.0213485475787765	chitin catabolic process
genital ducts	GO:0006811	3/47	95/9251	0.0495448016965787	monoatomic ion transport
genital ducts	GO:0045892	2/47	35/9251	0.0495448016965787	negative regulation of DNA-templated transcription
hemolymph	GO:0016485	3/56	28/9251	0.024073494837503	protein processing
hemolymph	GO:0006979	3/56	37/9251	0.024073494837503	response to oxidative stress
hemolymph	GO:0042744	2/56	10/9251	0.024073494837503	hydrogen peroxide catabolic process
hemolymph	GO:0021556	2/56	13/9251	0.0309328648772152	central nervous system formation
hindgut	GO:0006355	11/49	377/9251	0.000125175721594579	regulation of DNA-templated transcription
hindgut	GO:0097037	3/49	16/9251	0.00145295483037622	heme export
hindgut	GO:0030154	4/49	52/9251	0.0020282490930532	cell differentiation
hindgut	GO:1990573	2/49	13/9251	0.0187323035747442	potassium ion import across plasma membrane
hindgut	GO:0007189	2/49	14/9251	0.0187323035747442	adenylate cyclase-activating G protein-coupled receptor signaling pathway

hindgut	GO:0007186	4/49	144/9251	0.0450727054994688	G protein-coupled receptor signaling pathway
malpighian tubules	GO:0097037	5/98	16/9251	2.39727606743874e-05	heme export
malpighian tubules	GO:0006814	5/98	38/9251	0.00114526085091965	sodium ion transport
malpighian tubules	GO:0006820	4/98	40/9251	0.01346432492384	monoatomic anion transport
midgut	GO:0005975	51/215	203/9251	1.16037234839854e-37	carbohydrate metabolic process
midgut	GO:0006508	58/215	425/9251	4.8781674427562e-28	proteolysis
midgut	GO:0045490	13/215	13/9251	8.44163473719252e-21	pectin catabolic process
midgut	GO:1901642	9/215	18/9251	1.06946277359058e-09	nucleoside transmembrane transport
midgut	GO:0050790	7/215	11/9251	1.27712596619306e-08	regulation of catalytic activity
midgut	GO:0006629	12/215	78/9251	2.16071887361736e-06	lipid metabolic process
midgut	GO:0006820	8/215	40/9251	2.64210506294148e-05	monoatomic anion transport
midgut	GO:0016042	7/215	29/9251	2.64210506294148e-05	lipid catabolic process
midgut	GO:0006032	5/215	20/9251	0.000528262803880951	chitin catabolic process
ovaries	GO:0007275	10/139	129/9251	0.00175535527343945	multicellular organism development
ovaries	GO:0043161	8/139	126/9251	0.0178845629117829	proteasome-mediated ubiquitin-dependent protein catabolic process
ovaries	GO:0006511	9/139	161/9251	0.0178845629117829	ubiquitin-dependent protein catabolic process
ovaries	GO:0006749	4/139	30/9251	0.0190096981794287	glutathione metabolic process
testes	GO:0060271	32/427	60/9251	2.57159676827487e-25	cilium assembly
testes	GO:0003341	16/427	19/9251	1.94390917031109e-17	cilium movement
testes	GO:0007018	28/427	84/9251	1.09851922509659e-15	microtubule-based movement
testes	GO:0003254	9/427	16/9251	2.64461031339478e-07	regulation of membrane depolarization
testes	GO:0042073	6/427	11/9251	9.98585752314717e-05	intraciliary transport

testes	GO:0000226	10/427	43/9251	0.00045144610663387	microtubule cytoskeleton organization
testes	GO:0071805	11/427	57/9251	0.000967037529107494	potassium ion transmembrane transport
testes	GO:0007131	5/427	11/9251	0.00128297719258605	reciprocal meiotic recombination
testes	GO:0006099	9/427	41/9251	0.00128297719258605	tricarboxylic acid cycle
testes	GO:0035725	10/427	56/9251	0.00293753879490391	sodium ion transmembrane transport
testes	GO:0044782	5/427	14/9251	0.00371398302660593	cilium organization
testes	GO:0035556	12/427	89/9251	0.00884247039216909	intracellular signal transduction
testes	GO:0006096	7/427	35/9251	0.0100679957762013	glycolytic process
testes	GO:0009190	5/427	18/9251	0.0107136971000423	cyclic nucleotide biosynthetic process
testes	GO:0006334	4/427	11/9251	0.0107138699282137	nucleosome assembly
testes	GO:0007017	5/427	19/9251	0.0122455116905559	microtubule-based process
testes	GO:0006123	4/427	12/9251	0.0129074990638973	mitochondrial electron transport, cytochrome c to oxygen
testes	GO:0007283	4/427	12/9251	0.0129074990638973	spermatogenesis
testes	GO:0006006	4/427	13/9251	0.0170244765924613	glucose metabolic process
testes	GO:0036211	5/427	22/9251	0.0197884239111766	protein modification process

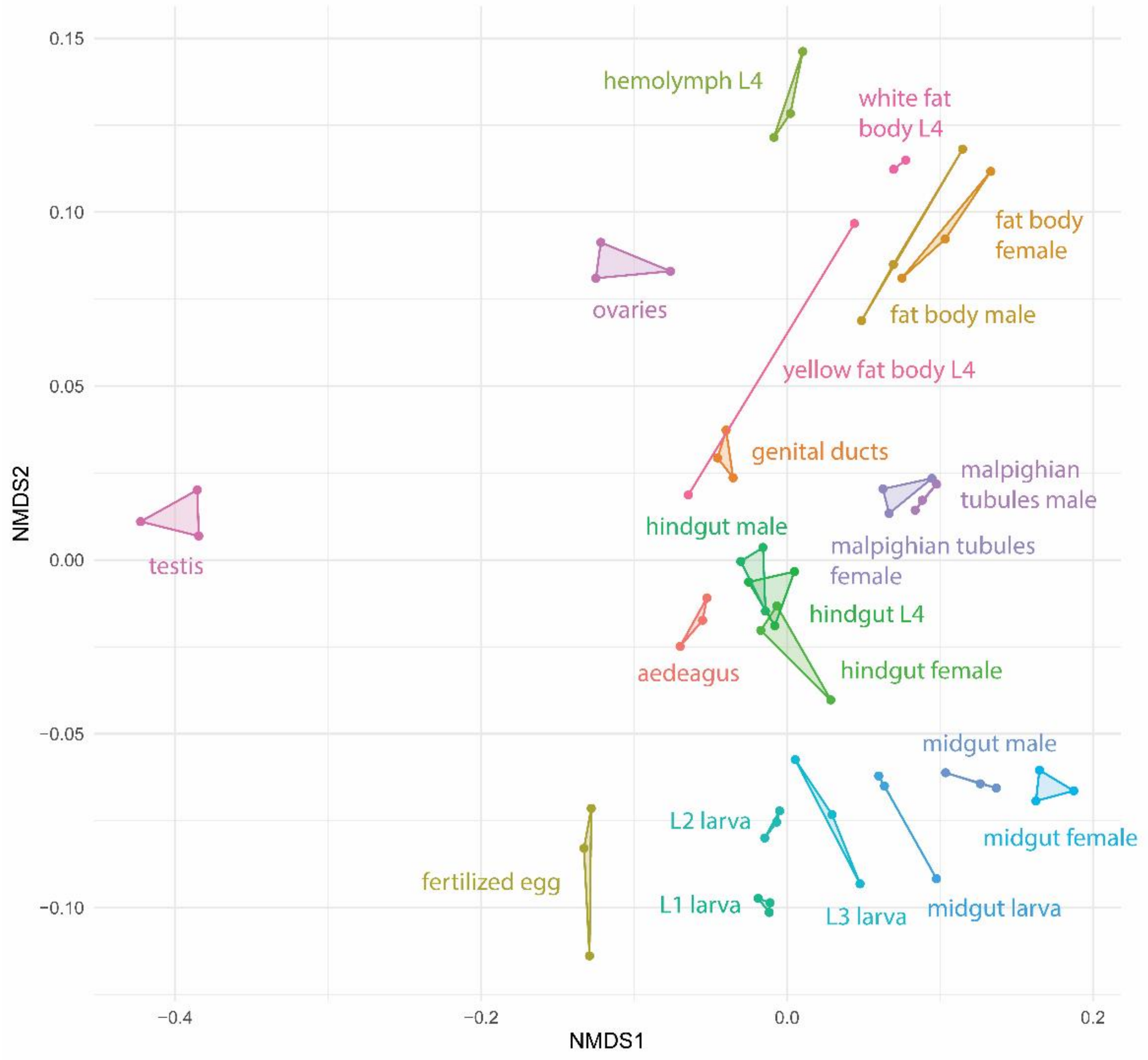

Supplementary figure 1. Non-metric multidimensional scaling (NMDS) of the samples based on log2(TPM+1) values. Biological replicates are connected. A yellow fat body of L4 appear clearly as an outlier, it has been removed from the dataset.

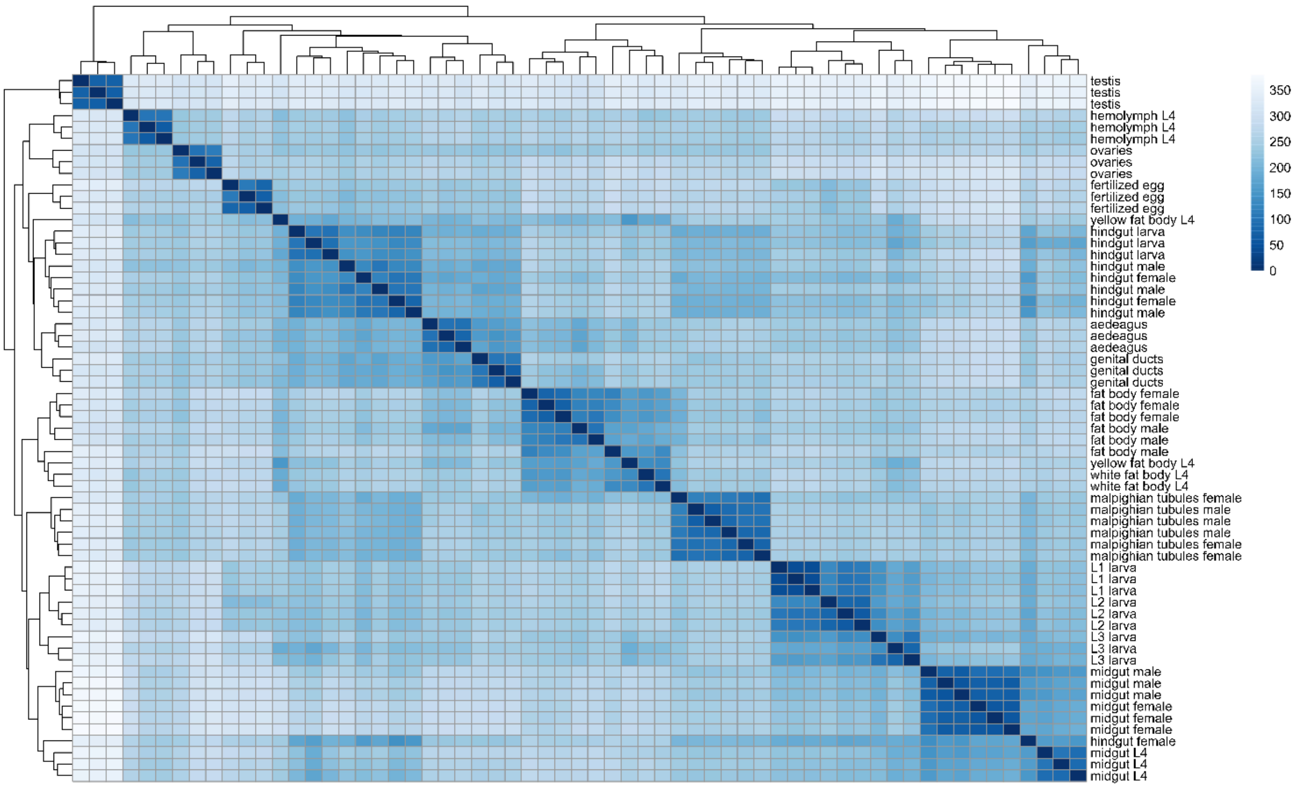

Supplementary figure 2. Heatmap showing the pairwise comparisons of log2(TPM) values across the 61 sequenced samples. A yellow fat body and a hindgut of female don't cluster with the replicates of the same type. Thy have been removed from the dataset.

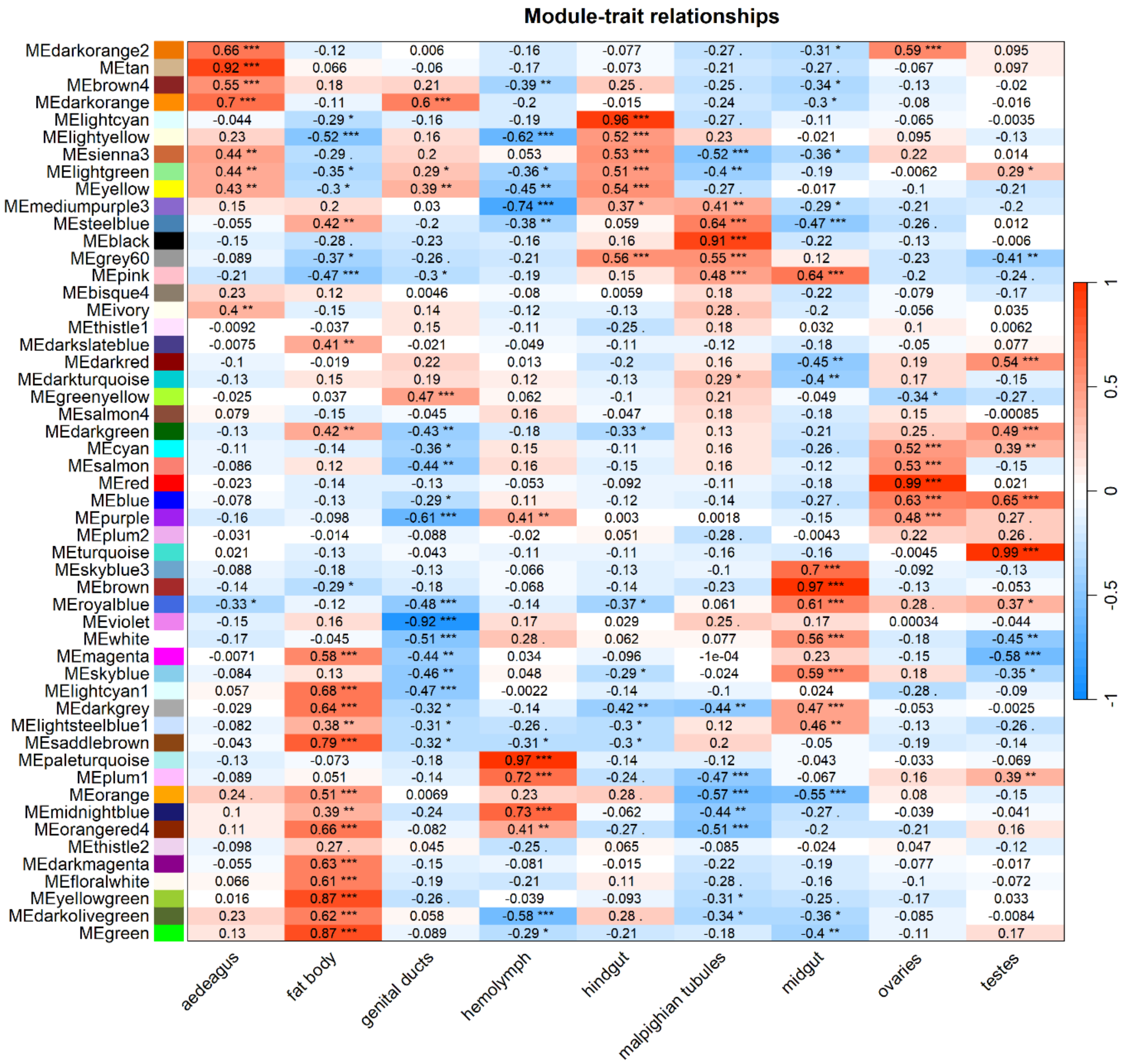

Supplementary figure 3. Pearson correlation between the modules and the tissues. The significance of the correlation is indicated with stars (p-value ≤ 0.001: ***, ≤0.01: **, ≤ 0.05: *, ≤0.1: .).
